# Characterization and evaluation of tissue-mimicking gelatin phantoms for use with MRgFUS

**DOI:** 10.1186/s40349-015-0030-y

**Published:** 2015-06-16

**Authors:** Alexis I. Farrer, Henrik Odéen, Joshua de Bever, Brittany Coats, Dennis L. Parker, Allison Payne, Douglas A. Christensen

**Affiliations:** Department of Bioengineering, University of Utah, Salt Lake City, UT USA; Utah Center for Advanced Imaging Research, Department of Radiology, University of Utah, Salt Lake City, UT USA; Department of Physics and Astronomy, University of Utah, Salt Lake City, UT USA; School of Computing, University of Utah, Salt Lake City, UT USA; Department of Mechanical Engineering, University of Utah, Salt Lake City, UT USA; Department of Electrical and Computer Engineering, University of Utah, Salt Lake City, UT USA

**Keywords:** Phantoms, Gelatin, MRgFUS, Tissue-mimicking

## Abstract

**Background:**

A tissue-mimicking phantom that accurately represents human-tissue properties is important for safety testing and for validating new imaging techniques. To achieve a variety of desired human-tissue properties, we have fabricated and tested several variations of gelatin phantoms. These phantoms are simple to manufacture and have properties in the same order of magnitude as those of soft tissues. This is important for quality-assurance verification as well as validation of magnetic resonance-guided focused ultrasound (MRgFUS) treatment techniques.

**Methods:**

The phantoms presented in this work were constructed from gelatin powders with three different bloom values (125, 175, and 250), each one allowing for a different mechanical stiffness of the phantom. Evaporated milk was used to replace half of the water in the recipe for the gelatin phantoms in order to achieve attenuation and speed of sound values in soft tissue ranges. These acoustic properties, along with MR (T_1_ and T_2_*), mechanical (density and Young’s modulus), and thermal properties (thermal diffusivity and specific heat capacity), were obtained through independent measurements for all three bloom types to characterize the gelatin phantoms. Thermal repeatability of the phantoms was also assessed using MRgFUS and MR thermometry.

**Results:**

All the measured values fell within the literature-reported ranges of soft tissues. In heating tests using low-power (6.6 W) sonications, interleaved with high-power (up to 22.0 W) sonications, each of the three different bloom phantoms demonstrated repeatable temperature increases (10.4 ± 0.3 °C for 125-bloom, 10.2 ± 0.3 °C for 175-bloom, and 10.8 ± 0.2 °C for 250-bloom for all 6.6-W sonications) for heating durations of 18.1 s.

**Conclusion:**

These evaporated milk-modified gelatin phantoms should serve as reliable, general soft tissue-mimicking MRgFUS phantoms.

## Background

Magnetic resonance-guided focused ultrasound (MRgFUS) is a promising, emerging technology that has been utilized as a thermal therapy with applications that include the treatment of uterine fibroids [1, 2], prostate tumors [3, 4], breast tumors [5, 6], essential tremor [7, 8], and bone palliative care [9, 10]. In MRgFUS studies, phantoms serve multiple needs, including quality assurance monitoring [11] and testing acoustic, mechanical, and thermal models [12, 13]. Phantoms can also be used to verify ultrasound exposure parameters, such as determining the specific absorption rate deposited, verifying beam patterns, and validating beam steering. Also, phantoms allow testing of magnetic resonance (MR) pulse sequences that are used to monitor MRgFUS treatments, to measure tissue displacement with acoustic radiation force imaging (ARFI), and for MR-temperature imaging.

There are several commercial phantom options available for MRgFUS therapies, such as those by ATS (ATS Laboratories Inc., Bridgeport, CT, USA) and CIRS (Computerized Imaging Reference Systems Inc., Norfolk, VA, USA). Commercial phantoms that are made consistently with controlled properties allow direct comparison of results from multiple groups that use the same product. Furthermore, commercial phantoms often have published values for several properties. While commercial phantoms are good options for many applications, their properties are not easily changed for specific applications. Custom making a phantom in-house provides an option for fabricating phantoms with shapes and properties tailored to the application and tissue of interest. The drawback in custom fabrication is the potential variability between batches, which may make experimental repeatability difficult. The purpose of this work was to fabricate a simple gelatin phantom suitable for both thermal ablation and ARFI testing and to characterize it by measuring its acoustic, MR, mechanical, and thermal properties, while also comparing it with soft tissue values of interest, such as in muscle, fat, breast, and brain tissues.

## Materials and methods

### Phantom fabrication

All phantoms were made using porcine gelatin powder of either 250-bloom (ballistics gelatin, Vyse Gelatin Co., Schiller Park, IL, USA), 175-bloom (Sigma-Aldrich Corp., St. Louis, MO, USA), or 125-bloom (Vyse). The bloom value is defined by the Gelatin Manufacturers Institute of America by measuring the weight (in grams) necessary to displace a 0.5-in diameter piston 4 mm into the solid gelatin, made with 6.67 % gelatin, 17 h after production and at a temperature of 10 °C [14]. Due to the strong relationship between bloom number and the mechanical properties of the phantom, in this paper we will refer to each phantom by its bloom number, where an increasing bloom value equates to an increased stiffness of the final gelatin solid.

The method for fabricating phantoms described herein was modified from the standard ballistics gelatin recipe [15] in that some of the water normally used was replaced with evaporated milk (Nestlé Carnation Evaporated Milk: Vitamin D Added) containing 6.3 % fat to add absorption and increase the speed of sound of the final phantoms. Evaporated milk was selected as the primary attenuation component for its widespread availability and its previous uses in agar-based phantoms [16].^1^ In this study, it was found that both the speed of sound and attenuation of the gelatin increased as the percentage of evaporated milk to water was increased during fabrication, as shown in Table 1. While the ratio of 70 % evaporated milk/30 % water produced the highest attenuation coefficient, its speed of sound was on the higher end of the soft tissue range. The 50 % evaporated milk/50 % water ratio produced a speed of sound in the mid-range for soft tissues with an attenuation value still within the range of soft tissues. Therefore, a ratio of 50 % water plus 50 % evaporated milk was used during fabrication for the phantoms characterized in this paper.

**Table 1:** Acoustic and mechanical property values for various percentages of water volume replaced with evaporated milk (250-bloom gelatin)

	Speed of sound [m/s] (*n* = 1)	Attenuation [dB/cm/MHz] (*n* = 1)	Young’s modulus [kPa] (*n* = 3)
0 % milk	1479	0.07	17.8 ± 0.7
30 % milk	1516	0.33	28.5 ± 1.2
50 % milk	1549	0.54	29.4 ± 4.7
70 % milk	1567	0.65	32.0 ± 0.8

To create each liter of the 50 % water/50 % milk gelatin phantom, 111 grams of gelatin powder with one of the three different bloom values (125, 175, and 250) is mixed with 225 mL of de-ionized degassed water in a sanitized container that needs to be capable of containing the final volume of the phantom. Though the standard definition of bloom is based on 6.67 % gelatin, we used the 11.1 % recommended by the gelatin manufacturer. Ten drops of Vyse defoamer solution (Vyse Gelatin Co., Schiller Park, IL, USA) are added after the powder and water have been mixed. In a separate sanitized container, 275 mL of degassed de-ionized water is mixed with 500 mL of evaporated milk. The evaporated milk/water mixture is heated to 80 °C, then poured into the gelatin/water mixture where it is thoroughly stirred until all the gelatin is fully dissolved, resembling a uniform liquid. This mixture is allowed to cool to 40 °C before pouring it through a strainer into the final (sanitized) phantom mold.

**Fig. 1:**
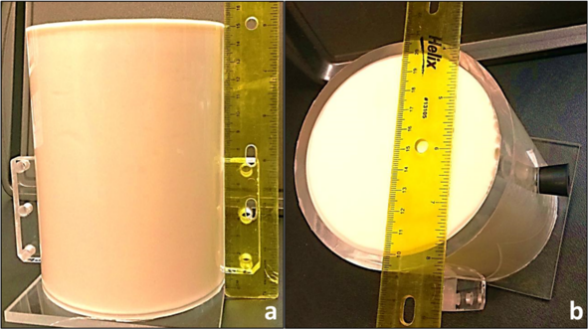
Typical gelatin phantom housed in our custom-built phantom holder. The holder is made of acrylic tubing **a** with a height of 15 cm and **b** with an inner diameter of 10 cm. A 0.1-mm film of clear PVC is adhered with silicone on both ends of the holder, creating an ultrasound transparent barrier

In our studies, no preservatives were added to the phantoms, but if desired, this could be added to the uniform gelatin/milk liquid mixture before it cooled to 40 °C. Once the gelatin was poured into its mold (an example is shown in Fig. 1), the mold was placed in a 10 °C refrigerator where it was stored until a few hours prior to use. The mold shown in Fig. 1 was designed to fit our MRgFUS system, allowing for consistent placement and positioning of the focal beam from phantom to phantom. It is an acrylic cylinder (10-cm inner diameter, 15-cm height, ~1.2-L volume) whose open ends are sealed with a thin film of ultrasound transparent clear PVC (~0.1-mm) adhered with silicone to create an air-tight seal. For all experimental values reported in this study, the phantoms were allowed to return to nominal room temperature (approximately 20 °C) prior to testing, unless otherwise noted. All testing was done within 3 to 18 h after each phantom was poured.

### Phantom characterization

#### Acoustic properties

**Fig. 2:**
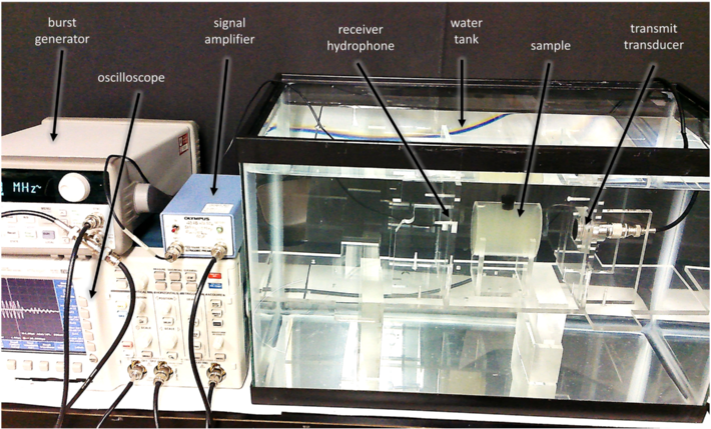
Setup for performing the through-transmission measurements used in calculating the speed of sound and attenuation of the samples

The speed of sound and attenuation coefficient of samples of each of the three bloom values were measured using the through-transmission technique [17, 18], as shown in Fig. 2, using phantoms housed in smaller holders made of acrylic tubing with a length of 7 cm and an inner diameter of 5.7 cm. Four different-frequency 5-cycle bursts were first transmitted and received with no sample in place (water only), at 0.6, 1.0, 1.8, and 3.0 MHz, then repeated with the phantom sample in place. A 1-MHz fundamental-frequency transmit transducer (Panametrics-NDT, V314, Waltham, MA, USA) was used that was broadband enough to cover the range of the three lower frequencies. Six different phantom samples were tested for each of the three bloom values, made by following the same recipe but fabricated at different times, for a total of 18 phantoms for acoustic property determination.

The speed of sound of each sample (${c}_s\Big)$ was found from

1 $$ {c}_s=\frac{c_w\cdot l}{l+{c}_w\Delta t}, $$

where *c*_*w*_ is the speed of sound of water and *l* is the length of the gelatin sample. The time delay Δ*t* between the two signals was found from the maximum of a cross-correlation of the analytic signals obtained by a Hilbert transform of the two received waveforms [17].

The attenuation ($\alpha$) of the sample was obtained from

2 $$ \alpha =\frac{-10{ \log}_{10}\left(\frac{{\displaystyle {\int}_{t_3}^{t_4}}{p_s}^2dt}{{\displaystyle {\int}_{t_1}^{t_2}}{p_w}^2dt}\right)}{l}, $$

where *p*_*w*_ is the pressure waveform for the water-only measurement and *p*_*s*_ is the pressure waveform when the sample is in place. The integrals were evaluated in the time domain by windowing the received waveform at each of the four selected frequencies (0.6, 1.0, 1.8, and 3.0 MHz) at the start of each waveform (*t*_1_, *t*_3_) and at the end (*t*_2_, *t*_4_), to isolate the sample from noise. A line was fitted to each attenuation value found at each frequency using linear least squares method in which the slope of the line provided the final attenuation value assigned to the sample in dB/cm/MHz.

#### MR properties

Phantom MR properties were measured on a Siemens 3T MRI scanner (TIM Trio, Siemens Medical Solutions, Erlangen, Germany). One phantom was tested for each bloom value using the mold shown in Fig. 1. The T_1_ and T_2_* values were obtained using short T_1_ inversion recovery (STIR) and multi-echo gradient recalled echo (ME GRE) sequences, respectively. For all measurements, care was taken to optimize the shimming. The imaging parameters for the STIR sequence were as follows: repetition time (TR) 6000 ms, echo time (TE) 17 ms, inversion recovery times (TI) between 50 to 2500 ms (8 different values), field of view (FoV) 200 × 128 mm, resolution (Res) 1.6 × 1.6 × 3.0 mm, flip angle (FA) 90/180°, and bandwidth (BW) 240 Hz/pixel. For the ME GRE sequence, the parameters were the following: TR 90 ms, TE between 2.83 to 80 ms (12 different values), FoV 192 × 144 mm, Res 0.8 × 0.8 × 5.0 mm, FA 20°, and BW 810 Hz/pixel.

A single-loop single-channel RF receive-only coil that was custom-built in-house for the phantom molds was used to detect the signal. The coil’s diameter was slightly larger than the 11.5-cm outer diameter of the cylindrical phantom holders (shown in Fig. 1) and was placed around the phantom 3 cm above the bottom edge of the phantom.

#### Mechanical properties

Young’s modulus for the gelatin phantoms was measured in unconfined compression with an Instron 5944 single-column testing system (Norwood, MA, USA). For mechanical property characterization, three batches were made for each gelatin-bloom value. Each batch of a given bloom value yielded multiple disk specimens uniformly created with a machined metal punch. Each disk had a diameter of 20 mm and a height of 10 mm; six sample disks were measured per batch. A frictionless boundary was achieved by applying canola oil to the surface of both compression plates. The top compression disk had a diameter of 22 mm, while the bottom plate’s diameter was 76 mm. A total strain of 15 % was performed (1.5-mm compression) on each disk in 2 s (strain rate, 0.075/s). Instron’s Bluehill 3 software was used to acquire the measured force and displacement, which in turn were used to calculate Young’s modulus. The Young’s modulus was averaged for the six disks in each batch, then averaged across the three batches. This was done for each bloom value (6 disks per batch, 18 disks per bloom value, 54 disks total).

Density was calculated by measuring the mass of each disk, then by measuring the volume of water displaced by each sample disk in a graduated cylinder. Six sample disks were measured from one batch for each of the three gelatin-bloom values, and then averaged to yield the density of each bloom value.

#### Thermal properties

Thermal diffusivity (*ψ*) and thermal conductivity (*k*) were determined with a KD2 Pro invasive thermal probe (Decagon Devices, Inc., Pullman, WA, USA). For each gelatin-bloom value, three measurements were obtained at different locations within a single phantom (selected from those used for acoustic measurements) and then averaged. The two-prong probe was inserted 3 cm into the phantom sample, where it remained undisturbed for approximately 3 min.

The specific heat capacity (*c*_*p*_) was calculated using Eq. 3 for each of the three bloom values. The average density (*ρ*) from the mechanical tests was used for each bloom value, but the three individual thermal diffusivity and thermal conductivity values measured for each sample with the KD2 Pro were used to calculate three separate specific heat capacities per bloom value, which were then averaged.

3 $$ {c}_p=\frac{k}{\rho \cdot \psi } $$

#### Thermal repeatability

Since these phantoms are intended to be used in heating studies with MRgFUS, it is important that they perform consistently through multiple heating cycles. Thermal repeatability testing was carried out on the three bloom-valued phantoms used for the MR-property determination by heating each phantom with a sequence of ultrasound exposures, interleaving lower power sonications with higher power exposures. During each exposure, 3D temperature maps were obtained with the proton resonant frequency (PRF)-shift MR-thermometry technique [19]. A 1-MHz 256-element phased-array transducer (Imasonic, Voray-sur-l’Ognon, France) with a focal distance of 13 cm (aperture diameter 14.5 cm, f-number 0.90) driven by electronics developed by Image Guided Therapy (Pessac, France) was employed for this testing. The heating parameters and order of each sonication for all blooms are provided in the next section. All powers provided were converted from electrical input watts to acoustic output watts using a calibration factor obtained with a radiation-force balance technique to measure the efficiency of the transducer. The 125-bloom and 175-bloom phantoms were exposed to fewer sonications based upon the experience with the 250-bloom phantom. Our initial thermal testing was performed on the 250-bloom phantom. We started at an initial low-power sonication of 6.6 W, then increased the power in small increments while interleaving with the 6.6-W low-power heating. By establishing the low- and high-power values and thereby setting the medium-power value, this allowed us to select fewer sonication powers for the 125-bloom and 175-bloom phantoms.

All heating was done with the geometric focus positioned 3 cm into the phantoms. A fiber optic temperature probe (Neoptix, Quebec, Canada) was inserted 4 cm into the other side of the phantom to measure the bulk temperature of the gelatin, approximately 8 cm away from the beam focus; all phantoms started at the MR suite’s ambient temperature (~24 °C).

The 3D MRI temperatures in the phantoms were obtained using a segmented GRE echo planar imaging (EPI) pulse sequence with TR 25 ms, TE 13 ms, FoV 192 × 96 × 32 mm, Res 1.2 × 1.2 × 2.0 mm (zero-filled interpolated to 0.5-mm isotropic voxel spacing), number of slices 16, FA 20°, BW 744 Hz/pixel, EPI factor 9, echo spacing 1.59 ms, acquisition time 3.625 s, with no fat saturation pulse applied. The start of each 18.125 s ultrasound-heating exposure was synchronized with the beginning of the sixth MR measurement using a fiber optic trigger pulse emitted by the pulse sequence. Between each heating exposure was a 10-min cooling period. Based on our previous experience with MR-temperature measurements in similar phantoms, the 10-min minimum-cooling interval was found sufficient to allow the heated region of the phantoms to return to a baseline-temperature value.

## Results

Table 2 summarizes the results of the acoustic, MR, mechanical, and thermal property measurements of the gelatin phantoms. Also shown are literature values for various soft tissues, specifically muscle, fat, breast, and brain tissue. All literature tissue values listed represent the tissue type as a whole, unless otherwise noted. Since there is considerable variation in the properties of tissues due to the heterogeneity of real tissue, we have selected a mid-range value to provide for comparison in Table 2. In Table 2, “*n*” refers to the number of times a test was performed within the same batch of gelatin, while “*N*” refers to the number of times a test was performed on separate batches of gelatin. The intra-batch standard deviation for T_1_, T_2_^*^, density, thermal diffusivity, and specific heat capacity is reported in Table 2, while the inter-batch standard deviation is reported for speed of sound, attenuation coefficient, and Young’s modulus values.

**Table 2:** Property values for gelatin phantoms and representative soft tissues (average ± 1 standard deviation)

Property	125-bloom gelatin phantom	175-bloom gelatin phantom	250-bloom gelatin phantom	Brain	Breast	Fat	Muscle
Speed of sound (m/s) (*n* = 1, *N* = 6)	1553 ± 21	1551 ± 15	1553 ± 10	1562 [30]	1510 [30]	1476_sc_ [30]	1582_sk_ [30]
Attenuation (dB/cm/MHz) (*n* = 1, *N* = 6)	0.50 ± 0.05	0.53 ± 0.08	0.54 ± 0.08	0.58 [30]	0.75 [30]	0.6_sc_ [30]	1.1_sk_ [30]
T_1_ @ 3T (ms) (*n* = 1^a^, *N* = 1)	970 ± 3	853 ± 3	1093 ± 5	1084_wm_ [40]	1445_gl_ [41]	367_br_ [41]	1412 [40]
T_2_ @ 3T (ms) (*n* = 1^a^, *N* = 1)	58 ± 7	55 ± 7	67 ± 12	65_wm_ [40]	22_gl_ [42]	68 [43]	42 [40]
Density (kg/m^3^) (*n* = 6, *N* = 1)	1067 ± 34	1058 ± 35	1057 ± 44	1041 [44]	1058 [44]	911 [44]	1090 [44]
Young’s modulus (kPa) (*n* = 6, *N* = 3^b^)	9.5 ± 1.8	18.8 ± 2.7	29.4 ± 4.7	0.5–6 [45–47]	22–76_gl_ [48]	12–26_br_ [48]	6–15_rst_ [30, 49, 50]
Thermal diffusivity (mm^2^/s) (*n* = 3^c^, *N* = 1)	0.144 ± 0.004	0.147 ± 0.004	0.143 ± 0.003	0.138 [30]	0.728_br_ [51]	0.0741 [51]	0.148 [30]
Specific heat capacity (J/kg/K) (*n* = 3^c^, *N* = 1)	3673 ± 159	3451 ± 97	3635 ± 88	3630 [44]	2960_gl_ [44]	2348 [44]	3421 [44]

*sc* subcutaneous fat, *sk* skeletal muscle, *wm* brain white matter, *gl* glandular breast tissue, *br* breast fat, *rst* at rest

^a^Mean and standard deviation obtained over voxels included within the ROI during one measurement

^b^Average of three batches per bloom value, six disks per batch

^c^Three measurements taken within the same phantom at different locations

### Thermal repeatability

**Fig. 3:**
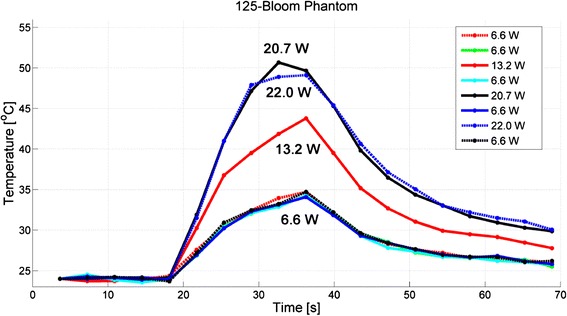
Thermal repeatability for the 125-bloom phantom, demonstrating the degree of consistency of achieving the same measured peak temperature for a given acoustic power. The order of applied power is shown in the legend

**Fig. 4:**
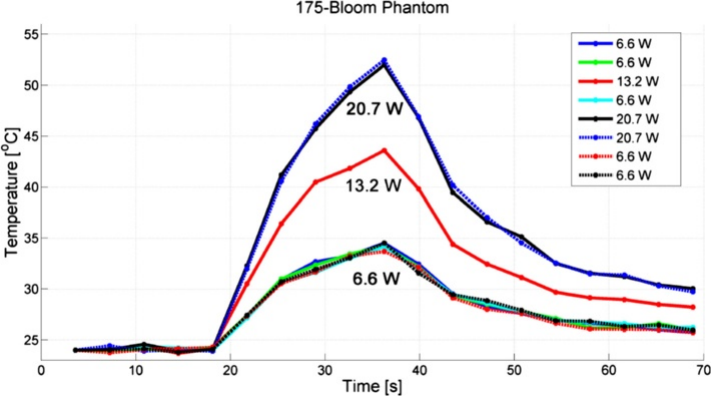
Thermal repeatability for the 175-bloom phantom, demonstrating the degree of consistency of achieving the same measured peak temperature for a given acoustic power. The order of applied power is shown in the legend

**Fig. 5:**
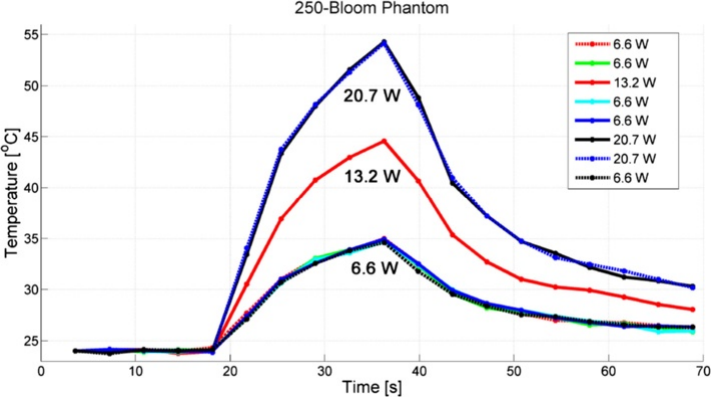
Thermal repeatability for the 250-bloom phantom, demonstrating the degree of consistency of achieving the same measured peak temperature for a given acoustic power. For clarity in this figure, only some representative runs are included. The order of applied power was *6.6*, *6.6*, 6.6, 10.1, 6.6, 13.2, 6.6, *13.2*, *6.6*, 15.4, 6.6, 17.6, *6.6*, *20.7*, *20.7*, and *6.6* W (italicized values reflect the runs shown in the figure and listed in the legend)

Thermal repeatability was tested for phantoms of each bloom value, and the temperature responses at the location of peak temperature for the 125-bloom phantom are displayed in Fig. 3, the 175-bloom phantom in Fig. 4, and the 250-bloom phantom in Fig. 5. Table 3 provides the average temperature rises (± one standard deviation) for all 6.6-, 13.2-, and 20.7-W heatings for all three gelatin-bloom values. The positions of the peak temperatures for all 32 sonications were located within the same scan-resolution voxel (1.2 × 1.2 × 2.0 mm), except for the 22.0-W sonication in the 125-bloom phantom, which was located 3 mm (approximately one voxel) deeper into the phantom, away from the transducer.

**Table 3:** Thermal repeatability of gelatin phantoms: peak-temperature rise

Gelatin phantom	Low-power heating (6.6 W)	Medium-power heating (13.2 W)	High-power heating (20.7 W)
125 bloom	10.4 ± 0.3 °C (*n* = 5)	19.8 °C (*n* = 1)	26.7 °C (*n* = 1)
175 bloom	10.2 ± 0.3 °C (*n* = 5)	19.6 °C (*n* = 1)	28.2 ± 0.2 °C (*n* = 2)
250 bloom	10.8 ± 0.2 °C (*n* = 9)	20.5 ± 0.1 °C (*n* = 2)	30.2 ± 0.1 °C (*n* = 2)

For the 125-bloom phantom, the five 6.6-W low-power exposures, interspersed with the higher power sonications, produced peak temperature increases of 10.4 ± 0.3 °C. The interspersed medium-power exposure of 13.2 W yielded a temperature rise of 19.8 °C, and the high-power exposure of 20.7 W had a temperature rise of 26.7 °C. To analyze the temperature-measurement uncertainty, the standard deviation through time (including all 16 time steps) for each of 20 × 20 × 20 voxels (i.e., a total of 8000 voxels) in an unheated region of the phantom was calculated. The mean and standard deviation of these 8000 standard deviation values was 0.20 ± 0.03 °C, demonstrating the good precision in the temperature measurements.

For the 175-bloom phantom, the five 6.6-W low-power exposures produced temperature increases of 10.2 ± 0.3 °C. The interspersed medium-power sonication of 13.2 W yielded a temperature rise of 19.6 °C, and the 20.7-W high-power sonications had a temperature rise of 28.2 ± 0.2 °C. The temperature standard deviation through time of an unheated 20 × 20 × 20 voxel region of the 175-bloom gelatin phantom was 0.23 ± 0.04 °C.

For clarity, not all sixteen heating runs performed in the 250-bloom phantom are shown in Fig. 5, but all nine run values are included in the results for the 6.6-W low-power temperature exposures given in Table 3; for these exposures, the temperature increase was 10.8 ± 0.2 °C. The interspersed medium-power sonications of 13.2 W yielded a temperature rise of 20.5 ± 0.1 °C, and the 20.7-W high-power sonications had a temperature rise of 30.2 ± 0.1 °C. The temperature standard deviation through time of an unheated 20 × 20 × 20 voxel region of the 250-bloom phantom was 0.17 ± 0.03 °C.

## Discussion

In this study, the acoustic, MR, mechanical, and thermal properties for gelatin phantoms with three bloom values have been characterized using independent methods. These easy-to-make gelatin phantoms provide properties of the same order of magnitude as soft tissues, without precisely mimicking a particular tissue. However, it is also important to acknowledge that each tissue type is not a homogeneous medium, and these phantoms cannot mimic the typical heterogeneity of a specific tissue type that has a range of valid values present. Despite this limitation, these gelatins potentially allow a user to customize the phantom further to match the particular homogeneous properties of a specific soft tissue.

The nominal requirements for soft tissue-like acoustic properties developed over the years by the ultrasound phantom research community [16, 20–24] include a speed of sound near 1540 m/s and a frequency-dependent attenuation coefficient near 0.5 dB/cm/MHz. Commercial phantom manufacturers, such as ATS [25] and CIRS [26, 27], provide materials whose acoustic properties range within these values, as shown in Table 4. Custom-made phantoms using agar and evaporated milk by Madsen et al. [16] have speeds of sound and attenuation coefficients that closely match these values (Table 4). Additionally, Table 4 shows that the measured properties of the gelatins manufactured by Hall et al. [28] and the complex hydrogel-based tissue-mimicking material for use as a FUS phantom developed by King et al. [29] are also near these values. The gelatin phantoms presented in this work have measured property values that similarly fall within the range of these commercial and custom-made research phantoms (Table 4), as discussed next.

**Table 4:** Comparison of the average properties of commercial and published phantoms with our gelatin phantoms

Manufacturer and phantom type	Speed of sound [m/s]	Attenuation [dB/cm/MHz]	Young’s modulus [kPa]
ATS hydrogel-based [25]	1540	n/a	n/a
ATS rubber-based [25]	1440–1460	0.48–0.52	n/a
CIRS hydrogels [26, 27]	1480–1600	0.45–0.75	3–48
Madsen agar with evaporated milk [16]	1541–1543	0.1–0.7	n/a
Hall gelatins [28]	1559–1600	n/a	3–126
King hydrogel-based^a^ [29]	1539–1583	0.51–0.55	n/a
Our gelatins with evaporated milk:			
125-bloom	1532–1574	0.45–0.55	8–11
175-bloom	1536–1566	0.45–0.61	16–22
250-bloom	1543–1563	0.46–0.62	24–34

Values are given as a range of minimum to maximum, or as ±1 standard deviation of the mean as reported in the literature or on the company’s website

*n/a* not available

^a^King’s hydrogel phantoms also had a thermal diffusivity of 0.15 mm^2^/s and a calculated specific heat capacity of 3648 J/kg/K, which are comparable to the values of our measurements in Table 2

The top two rows of Table 2 provide representative acoustic literature values for several soft tissues (brain, breast, fat, and muscle) [30] along with averaged acoustic values measured in this study for the three different bloom-valued phantoms. The soft tissue values generally represent the tissue type as a whole, but the fat values represent subcutaneous fat and the muscle values are for skeletal muscle. The gelatin phantoms described in this paper match most closely with brain and muscle for speed of sound, but the attenuation values are at the low end of the spectrum, near that of fat and brain. A possible modification to the gelatin recipe to increase the attenuation is the addition of graphite powder [22, 31], which was not done in the present study. It is important to point out that any additions to adjust for a particular property may alter another property. For instance, the addition of graphite powder has been shown to increase Young’s modulus for gelatin phantoms used in Hall et al. [28].

Also in Table 2 are representative literature values for MR properties at 3T for brain, breast, fat, and muscle along with the average of the measured MR values for the three gelatin phantoms. The T_1_ and T_2_* literature values were measured in white matter for the brain and in glandular tissue for the breast. The fat T_1_ literature value was measured in breast fat. All three of the gelatin values have similar MR properties that fall between the literature values for the listed soft tissues. The 250-bloom gelatin phantom has both a T_1_ value and a T_2_* value very similar to brain white matter. If desired, the T_1_ values of the phantoms can be decreased by adding various amounts of copper sulfate to the gelatins during construction [32], though this may affect other properties as well.

Table 2 also compares the average of the measured mechanical properties for the three phantoms (density and Young’s modulus) with representative literature values for brain, breast, fat, and muscle. With the exception of fat, the gelatin phantoms and soft tissues have similar densities that are all slightly denser than water. Since the bloom value corresponds to the stiffness of the gelatin, the three phantom types exhibited different Young’s modulus values. The 125-bloom phantom had a Young’s modulus near that of brain and relaxed muscle. The 175-bloom gelatin’s Young’s modulus value was in the range of fat, while the 250-bloom gelatin best represents more stiff soft tissues, such as breast glandular tissue. It is important to note that the addition of the evaporated milk increased the gelatin’s Young’s modulus values, as shown in Table 1. Gelatin with a lower bloom value may be used to achieve a lower Young’s modulus, as well as using less gelatin powder during the construction of the phantom. To achieve a higher Young’s modulus value, cross-linkers such as formaldehyde and glutaraldehyde may be incorporated into the gelatin phantom-making process [33–36]. It can be hypothesized that the addition of cross-linkers would cause an increase in the speed of sound, since more stiff tissues tend to have a higher speed of sound. This could be measured by performing through-transmission testing on gelatins with a cross-linking agent added.

The last two rows of Table 2 compare representative literature values for the thermal properties of brain, breast, fat, and muscle along with the average of the measured values for the gelatin phantoms. The breast thermal diffusivity literature value is for breast fat, while the specific heat capacity is for glandular breast tissue. All three phantoms have similar thermal diffusivity and specific heat capacity values, and are similar to the literature values for brain and muscle. These values are somewhat higher than those reported for fat and breast tissue.

These gelatin phantoms demonstrated thermal repeatability when heated with MRgFUS using low-power heatings interspersed with medium- to high-power heatings, as summarized in Table 3 and shown in Figs. 3, 4, and 5. We judged the phantom thermally repeatable if both subsequent low- and high-power heatings produced peak temperatures consistent with the previous temperatures achieved at those powers (as evidence by the low standard deviation of the measured peak temperatures) and the position of the peaks occurred within the same scan-resolution voxel. Each of the three different bloom phantoms demonstrated repeatable temperature increases of 10.4 ± 0.3 °C for 125-bloom, 10.2 ± 0.3 °C for 175-bloom, and 10.8 ± 0.2 °C for 250-bloom for all low-power 6.6-W sonications, giving coefficients of variation (ratio of standard deviation to the mean) of 3, 3, and 2 % for the 125-bloom, the 175-bloom, and the 250-bloom gelatins, respectively. These low-power peak-temperature values along with the precision of our MR-temperature measurements demonstrate the high thermal repeatability of these gelatin phantoms. The small coefficient of variation indicates that temperature measurements in heating experiments are sufficiently repeatable in these phantoms to detect very small (sub-degree) temperature variations as significant with a small number of measurements.

The thermal repeatability tests showed that the magnitude and position of the temperature rise was predictable in 31 of 32 cases. Only for the highest power exposure (22.0 W) with the 125-bloom sample was there a deviation from the time and temperature patterns seen in previous exposures. The location of the peak temperature also shifted one voxel deeper into the phantom than measured in all other exposures. This may be due to spot melting of the gelatin at the focus; however, we did not do independent verification of this. Future studies will investigate the effects that cross-linkers have on the gelatin phantoms by re-testing their thermal repeatability. By adding cross-linkers to the gelatin, the temperature at which the gelatin phantoms melt can be increased [35, 37]. Though cross-linkers are known to increase the stiffness and melting temperature of gelatins and polymers, the effects that cross-linking will have on the density, thermal diffusivity, specific heat capacity, attenuation, speed of sound, and MR properties (T_1_ and T_2_*) for these gelatin phantoms will need further study.

Our group has employed the homogeneous soft tissue-mimicking phantoms described in this work for both thermal ablation evaluations and ARFI. All three bloom values with their varying Young’s moduli are useful for ARFI experiments and have been used in validating ARFI simulations with good correlation to experimental results [12]. Also, phantoms similar to the ones in this study have served our group as long-term quality-assurance phantoms (4 months) by adding a preservative (DOWICIL, Dow, Midland, MI, USA) during their fabrication. In addition, these gelatin phantoms have been used in complex molds with embedded ex vivo tissues, such as molding the gelatin around a skull cap [38] and embedding a kidney within the gelatin for a flow phantom [39].

## Conclusions

This work provides a general soft tissue, repeatable gelatin-phantom recipe that is easy to manufacture. Several of these simple gelatin phantoms have been characterized for acoustic, MR, mechanical, and thermal properties, and have been shown to demonstrate thermal repeatability. Future studies will entail adding cross-linkers to these phantoms to increase their melting temperatures and investigate the effect on other phantom properties. Also, future work can investigate the interrelationship between other additions and subsequent changes to the phantom properties. By exploring such modifications to the gelatin recipe, one may be able to adjust the gelatin to better match a specific property for a soft tissue of interest.

## Endnote

^1^Vegetable glycerin was also tested to replace water volume in the gelatin recipe in order to increase the phantom’s viscosity as a potential option for increasing the attenuation. However, the speed of sound increased more drastically than with evaporated milk with only a small increase in attenuation. Also, the glycerin was more difficult to keep degassed as air bubbles were visible in the final solidified gelatin phantoms.
